# Altered Hypercoagulability Factors in Patients with Chronic Chagas Disease: Potential Biomarkers of Therapeutic Response

**DOI:** 10.1371/journal.pntd.0004269

**Published:** 2016-01-04

**Authors:** Maria-Jesus Pinazo, Elizabeth de Jesus Posada, Luis Izquierdo, Dolors Tassies, Alexandre-Ferreira Marques, Elisa de Lazzari, Edelweiss Aldasoro, Jose Muñoz, Alba Abras, Silvia Tebar, Montserrat Gallego, Igor Correia de Almeida, Joan-Carles Reverter, Joaquim Gascon

**Affiliations:** 1 ISGlobal, Barcelona Centre for International Health Research (CRESIB), Hospital Clínic- Universitat de Barcelona, Barcelona, Spain; 2 Hemotherapy and Hemostasis Department, Hospital Clínic de Barcelona, Barcelona, Spain; 3 Border Biomedical Research Center, Department of Biological Sciences, University of Texas at El Paso, El Paso, Texas, United States of America; 4 Universidade Federal de Minas Gerais, Departamento de Parasitologia, Belo Horizonte, Minas Gerais, Brazil; 5 Health Biostatistics, ISGlobal, Barcelona Centre for International Health Research (CRESIB), Hospital Clínic- Universitat de Barcelona, Barcelona, Spain; 6 Laboratori de Parasitologia, Facultat de Farmàcia, Universitat de Barcelona, Barcelona, Spain; 7 Barcelona Institute for Global Health, Hospital Clínic—Universitat de Barcelona, Barcelona, Spain; FIOCRUZ - Minas, BRAZIL

## Abstract

Thromboembolic events were described in patients with Chagas disease without cardiomyopathy. We aim to confirm if there is a hypercoagulable state in these patients and to determine if there is an early normalization of hemostasis factors after antiparasitic treatment. Ninety-nine individuals from Chagas disease-endemic areas were classified in two groups: G1, with *T*.*cruzi* infection (n = 56); G2, healthy individuals (n = 43). Twenty-four hemostasis factors were measured at baseline. G1 patients treated with benznidazole were followed for 36 months, recording clinical parameters and performance of conventional serology, chemiluminescent enzyme-linked immunosorbent assay (trypomastigote-derived glycosylphosphatidylinositol-anchored mucins), quantitative polymerase chain reaction, and hemostasis tests every 6-month visits. Prothrombin fragment 1+2 (F1+2) and endogenous thrombin potential (ETP) were abnormally expressed in 77% and 50% of infected patients at baseline but returned to and remained at normal levels shortly after treatment in 76% and 96% of cases, respectively. Plasmin-antiplasmin complexes (PAP) were altered before treatment in 32% of G1 patients but normalized in 94% of cases several months after treatment. None of the patients with normal F1+2 values during follow-up had a positive qRT-PCR result, but 3/24 patients (13%) with normal ETP values did. In a percentage of chronic *T*. *cruzi* infected patients treated with benznidazole, altered coagulation markers returned into normal levels. F1+2, ETP and PAP could be useful markers for assessing sustained response to benznidazole.

## Author Summary

The manuscript describes the results of a study whose aim was to assess the tendency to coagulate in people suffering from a parasitic infection frequent in Latin America named *T*. *cruzi* infection or Chagas disease, by the study of several coagulation factors. According to the state of the art in this topic, specific treatment for Chagas disease is recommended in recent (acute) and late (chronic) stages of the infection. The effectiveness of current available drugs in the chronic stage of infection is still a topic of debate due to inconsistent results across studies and a lack of early measurable parameters of response to specific treatment. Another aim of this study was to determine if the presence of an upregulated procoagulative activity in plasma in people suffering *T*. *cruzi* infection could be used as potential marker that indicates therapeutic response in people at chronic stage of the disease. The results of this study suggest that measurements of alterations of procoagulative activity may be useful to indicate specific treatment for *T*. *cruzi* chronically infected patients and new data concerning early response to treatment biomarkers.

## Introduction

Chagas disease (CD) is one of 17 neglected tropical diseases recognized by the World Health Organization. Caused by the protozoan parasite *Trypanosoma cruzi*, it mainly affects people with poor socioeconomic status and limited health care access in endemic and nonendemic countries. [1, 2]

Thrombosis is considered as a pathological deviation of haemostasis, and it is characterized by intravascular thrombus formation and vessel occlusion. Perturbation of hemostasis is an important factor in the pathogenesis of thromboembolic events, which can be caused by blood flow dysregulation, endothelial injury, and coagulation system alterations.

Recently, is has been described that under certain circumstances thrombosis is a physiological process that constitutes an intrinsic effector mechanism of innate immunity, and the process has been defined as “immunothrombosis”. [3] It is activated after the recognition of pathogens and damaged cells, and inhibits pathogen dissemination and survival. Immunothrombosis can therefore be regarded as a newly identified, crucial element of intravascular immunity, which is a part of the immune system that encompasses a wide range of host strategies to detect and protect against pathogens in the vasculature. Dysregulation of immunothrombosis is likely to constitute a key event in the development of thrombotic disorders. [3]

Infectious disease can cause a hypercoagulable state through the upregulation of tissue factor in monocytes, the generation of procoagulant microparticles, the activation of the coagulation intrinsic pathway, platelet activation, and NETs (Neutrophil Extracellular Traps) release.[3] Different infectious agents may cause different responses but a final degree of hypercoagulability can be a common trait as one of the biological endpoints. Additionally, patients with chronic inflammation may also present platelet adhesion events, which are considered inflammatory processes and can be observed in patients with chronic *T*. *cruzi* infection, even in the asymptomatic stages. [4] Infection itself can cause vasculitis, increasing proinflammatory cytokine levels and perpetuating the risk of thrombotic events. [5] In the case of the Chagas’ disease the effect of hemostasis in the bradikinin formation, through the effect of factor XII activation in the Kallikrein-Kinin system, can modify the type 1 immune response and then modulate the antiparasite immunity as suggested in a mice model of subcutaneous infection by *T*.*cruzi*. [6]

Thromboembolic events and dilated cardiomyopathy, ventricular aneurysms, and intracavitary thrombosis are associated with CD. [7, 8] Rheological factors can induce intraluminal thrombus formation with the risk of embolism. [9] Alterations of molecular markers of coagulation system activation have been described in *T*. *cruzi* infection individuals with or without clinical thrombosis. [9–12] Other factors, such as injury to vessel walls by parasites or changes in blood viscosity due to host immune response, may influence in the development of thromboembolic events in *T*. *cruzi*-infected individuals without Chagas cardiomyopathy or other vascular risk factors. [13]Based on studies performed in humans with chronic *T*. *cruzi* infection, there are controversial results regarding the existence of a prothrombotic status in *T*. *cruzi*-infected patients. [13,14] There is an study in which a of higher prothrombotic status in the CD group was not found, but the control group were individuals without *T*. *cruzi* infection and heart failure. [14] In previous studies performed in murine models, several abnormalities of the heart microcirculation of individuals with chronic CD were pointed out, but they did not find evidence of thrombi and neither thromboembolism. [15, 16] Higher levels of the hypercoagulability markers prothrombin fragment 1+2 (F1+2), thrombin-antithrombin complexes (TAT), fibrinogen/fibrin degradation products, plasminogen activator inhibitor type 1 (PAI-1), and D-dimer have been reported in *T*. *cruzi*–infected patients compared with healthy individuals. [10, 11] A pilot study performed by our group showed that endogenous thrombin potential (ETP) and F1+2 levels were outside normal ranges in 73% and 80% of *T*. *cruzi*–infected patients without advanced heart disease, respectively. [12] We demonstrated a 100% and 73% decrease in these levels six months after treatment with benznidazole. Thus, if they prove to remain stable in time, hypercoagulability factors could be used as biomarkers of therapeutic response in CD. Besides, although whether or not chronic Chagas disease is an independent vascular risk factor remains to be confirmed. [17,18]

While specific treatment is recommended in both acute and chronic stages of infection [19,20], there are only two drugs (i.e., benznidazole and nifurtimox) available for the treatment of CD. The mechanism of action of benznidazole relates to the nitro-reduction of components of the parasite, the binding of metabolites of the nuclear DNA and k-DNA of *T*. *cruzi* and the lipids and proteins of the parasite. [21] In adults, benznidazole has a high rate of adverse effects, which can be classified into three groups: (i) hypersensitivity, including dermatitis with cutaneous eruptions (usually appearing between days 7 and 10), myalgias, arthralgias, and lymphadenopathy; (ii) polyneuropathy, paresthesias, and polineuritis usually during the 4th week of treatment); and (iii) bone marrow disorders, such as thrombopenic purpura and agranulocytosis (usually after the second week of treatment). [22]Furthermore, the effectiveness of these drugs in the chronic stage of infection is still a topic of debate due to inconsistent studies’ results [23–25] and a lack of early biomarkers of response to specific *T*. *cruzi* treatment with benznidazole. [26]

Following on from our pilot study [12], here we increased the sample size and extended follow-up to further investigate the value of hypercoagulability factors as biomarkers of treatment response in CD. We also added current treatment response parameters measured by conventional serology, serology for lytic anti-α-galactosyl (anti-α-Gal) antibodies against *T*.*cruzi* [27–29], and quantitative reverse transcription polymerase chain reaction (qRT-PCR). [30]

The aims of the study were to investigate alterations of hypercoagulability factors in patients chronically infected with *T*. *cruzi* and determine whether there is an early and sustainable improvement of the hypercoagulability factors after antiparasitic treatment.

## Methods

### Ethics Statement

Written informed consent was obtained from participants before being recruited (all of them were adults). Approval for the protocols and for the informed consent was obtained from the Hospital Clínic of Barcelona Ethics Review Committee.

### Design and Setting

This is a descriptive study of 99 individuals (56 with *T*.*cruzi* infection and 43 healthy individuals) from Latin American, where CD is endemic. All the individuals were evaluated at the Centre for International Health at Hospital Clínic in Barcelona, Spain.

### Recruitment and Participants

Ninety-nine individuals from CD-endemic areas living in Barcelona were invited to participate. Inclusion criteria were an age of over 18 years and provision of signed informed consent. Exclusion criteria were pregnancy, non-Chagasic cardiopathy, late chronic cardiac or digestive forms of CD, other acute or chronic infections, inflammatory or immunological diseases, and chronic systemic diseases (high blood pressure and diabetes).

### Procedures

After signing the informed consent form, participants were asked for clinical and epidemiological data, including area of origin and risk factors for the CD transmission. The information recorded included vascular risk factors, toxic habits, and cardiological and/or vascular events.

Conventional serology of *T*.*cruzi* infection was established using two ELISA kits: a commercial kit with recombinant antigens (BioELISA Chagas, Biokit S.A.,Barcelona-Spain) and an in-house kit with whole *T*.*cruzi* epimastigote antigen, as described. [12, 31]. Diagnosis was confirmed by a positive result on both tests. [19] Following serological tests results, participants were divided into two groups: those with *T*.*cruzi* infection (Group 1 [G1]) and those without (Group 2 [G2]). All the participants underwent human immunodeficiency virus testing, basic blood and biochemical tests (including renal and liver function), and specific evaluation of hemostasis factors.

For the hemostasis studies, blood was collected in citrate-containing tubes (Becton Dickinson), samples were centrifuged, and platelet-poor plasma aliquots were frozen at –80°C until assayed. Prothrombin time, activated partial thromboplastin time, coagulation factor VIII, protein C activity, free and total protein S levels, antithrombin and plasminogen activity, F1+2, plasmin-antiplasmin complexes (PAP), factor VIIa, PAI-1, P-selectin, factor V Leiden and prothrombin gene G20210A mutation, lupus anticoagulant and anticardiolipin antibodies were measured as previously described. [12] D-dimer was measured using an automated turbidimetric test (Siemens Healthcare Diagnostics) and ETP was assessed using a continuous chromogenic thrombin generation assay and ETP Curves software (Siemens). The ETP coagulation test was initiated by using human recombinant tissue factor, phospholipids, and calcium ions. ADAMTS-13 was measured using a commercial chromogenic method (American Diagnostica). Factor XIIa was determined by a direct quantitative commercially available immunoassay (Shield Diagnostics) with a highly specific monoclonal antibody that does not recognize its zymogen factor XII.[32] Plasma tissue factor levels were determined using a commercial kit (American Diagnostica) according to the manufacturer’s protocol. Plasma levels of von Willebrand factor antigen were determined by enzyme-linked immunosorbent assay (ELISA) (Corgenix). Procoagulant activity of microparticles was measured using a functional assay with the addition of factors Xa, Va, and prothrombin after microparticle capture in the solid phase using annexin V (Hyphen Biomed). Soluble CD40L was measured by ELISA (R&D Systems).

qRT-PCR [30] and a chemiluminescent ELISA assay based on a highly purified, trypomastigote-derived glycosylphosphatidylinositol-anchored mucin (tGPI-mucin) antigen for the serological detection of lytic anti-α-Gal antibodies against *T*.*cruzi* (AT CL-ELISA) [27–29, 33–36], were performed in G1 at month 0 (baseline), and 6, 12, 18, 24, 30, and 36 months post-treatment. For AT CL-ELISA, a serum sample was considered positive when the titer was ≥1.0 and negative when it was ≤0.9. Inconclusive or equivocal results were determined by a titer between 0.9 and 1.0. [27, 35]All sera were tested in duplicate and the results were expressed as the mean of two simultaneous determinations.

G1 patients were studied using a protocol that included a 12-lead electrocardiogram, chest X-ray, and echocardiogram. They were followed up every 6 months for at least 36 months. At each visit, clinical data were collected and the following tests were performed: ELISA, AT CL-ELISA, qRT-PCR, and hemostasis tests. Other tests were performed according to individual symptoms. Specific treatment with benznidazole (5 mg/kg/day for 60 days) was offered to all *T*.*cruzi*–infected patients, and those treated were monitored fortnightly for clinical and analytical assessment. Treatment was considered complete when at least 80% of the total dose was reached.

A hypercoagulable state is defined as the presence, in certain individuals, of thrombotic potentialities that activate the endothelium and the formative elements of the blood (mainly, platelets) that favors plasma kinetics that lead to the formation of thrombin, which disturbs fibrinolytic activity and produces hemorheological changes with turbulence phenomena that predispose to thrombogenesis. [18]

### Statistical Analysis

Quantitative variables were presented as medians and interquartile range (IQR) and were compared between groups using the Wilcoxon rank sum test. Qualitative variables were reported using absolute frequencies and percentages and between-group comparisons were made using Fisher’s exact test. Hypercoagulability biomarker variation over time was assessed using a mixed-effect linear regression model with a random intercept structure. Hypercoagulability factors were used as dependent variables and follow-up time as the explanatory variable, with one category for each time point: baseline, month 6 (reference for comparisons), and months 12, 18, 24, and 36. This type of model allows for the inclusion of random effects in addition to the overall error term. Random intercept regression was also used to assess whether antibody levels measured by ELISA and AT CL-ELISA approached the negative threshold during follow-up. The response variable was the distance from this threshold (i.e., the difference between each ELISA or AT CL-ELISA value and the negative cutoff) and the explanatory variable was the follow-up time from month six (reference) to month 36. The regression coefficients express the effect estimate of follow-up on the outcome variable.

The pattern of the relationships between hypercoagulability biomarkers was assessed by multiple correspondence analysis (MCA) using the Burt matrix approach. [37, 38] The MCA represents a method for analyzing multi-way contingency table containing measure of correspondence between row (subjects) and columns (levels of variables). The interpretation is based upon proximities between levels of variables (or points) in a low-dimensional map. The firsts dimensions (usually one or two) account for meaningful amounts of variance and are those retained for the map definition and interpretation. The first dimension accounts for a maximal amount of total variance in the observed variables. Under typical conditions, this means that the first component will be correlated with at least some of the observed variables. The second dimension has two important characteristics: it accounts for a maximal amount of variance in the data set that is not accounted for by the first dimension, thus it is correlated with some of the observed variables that not display strong correlations with dimension 1; and it is uncorrelated with dimension 1. Looking at the map, the proximity between levels of different variables means that these levels tend to appear together in the observations. Since the levels of the same variable cannot occur together, the proximity between levels of the same variable means that the groups of observations associated with these levels are themselves similar. A level far away from the origin (of the dimensions) means that is well-represented in the map, thus that level is meaningful for the interpretation of the dimension(s). All levels that are not useful for the solution are near the origin. Supplementary (passive) variables are those not used for the solution but mapped in the graph in order to help in the interpretation.

The biomarkers were classified into three categories: normalization of values throughout follow-up, non-sustained normalization during follow-up and normal values at baseline. Two additional variables were considered: qRT-PCR results during follow-up (categories: always negative and sometime positive) and level of adherence (categories: 80% and 100%). All the tests were 2-tailed and the confidence level was set at 95%. The analyses were performed using Stata 13 (Stata Corporation, College Station, TX, USA).

## Results and Discussion

Ninety-nine individuals (76 women) were studied. Fifty-six of these (43 women) were *T*.*cruzi–*positive (G1) and 43 (33 women) were *T*.*cruzi*–negative. The mean ages were 34 (SD, 9) years for the overall group (range 17–56, median 33), 37 (SD, 9) years for G1, and 32 (SD, 7) years for G2. Fifty G1 patients were treated with benznidazole (six were lost to follow-up before starting treatment due to unexpected work-related changes in the migratory process). Forty-five (90%) completed treatment. Eighty-six participants (87%) (51 [91%] in G1 and 35 [81%] in G2) were from Bolivia. None of the participants traveled to their countries or other CD-endemic areas during follow-up. The clinical and demographic data are summarized in Table 1. The epidemiological and baseline clinical data were similar in both groups, making them statistically comparable.

**Table 1:** Epidemiological data, vascular risk factors, and cardiovascular events in healthy and *T*. *cruzi*-infected individuals.

		Group 2: Healthy Individuals *n* (%)	Group 1: *T*. *cruzi*-Infected Patients (Baseline) *n* (%)
**Country of origin**	Bolivia	35 (81)	51(91)
	Argentina	1 (2)	2(4)
	Brazil	1 (2)	1 (2)
	Colombia	3 (7)	0
	Ecuador	2 (5)	1 (2)
	Paraguay	0	1 (2)
	Peru	1 (2)	0
**Toxic habits**	Smoking	1 (2)	1 (2)
	Alcohol intake	5 (12)	1 (2)
**Vascular risk factors**	High blood pressure	0	0
	Hyperlipidemia	4 (9)	5 (9)
	Diabetes mellitus	0	0
**Cardiovascular events**	Atrial fibrillation	0	0
	Valvulopathy*	0	4 (8)
	Cardiac failure	0	0
	Myocardial ischemia	0	0
	Stroke	0	0

Comparison of the 24 hypercoagulability biomarkers at baseline between (untreated) G1 and G2 individuals showed statistically significant differences for D-dimer (*P* = .0262); F1+2 (abnormal values in 43/56 G1 patients [77%], *P* < .0001), PAP (abnormal values in 17/56 G1 patients [30%], *P* = .0111), P-selectin (abnormal values in 7/56 G1 patients [13%] *P* = .0177), and ETP (abnormal values in 28/56 G1 patients [50%], *P* < .0013), and circulating microparticles (*P* = .0112) (Table 2). D-dimer levels were normal in all the individuals in G1 and G2, and microparticles were within the normal range in a high percentage of patients (86% in G1 and 93% in G2, *P* = .3402). Our findings showed that a high percentage of patients with chronic *T*.*cruzi* infection have a hypercoagulable state regardless the clinical stage of disease, thus confirming the observations of previous studies. [11–13]

**Table 2:** Descriptive analysis and comparisons of hemostasis parameters between pretreatment Group 1 (N = 56) and Group 2 (N = 43).

VARIABLE		Group 1 Median (IQR) [n]	Group 2 Median (IQR) [n]	*P* Value ^*a*^	Normal range (units)
D-dimer		228.5 (119.0) [56]	201.0 (125.0) [43]	0.0262	50–400 (μg/L)
Prothrombin fragment 1+2		**1.8 (1.3) [56]**	**0.8 (0.4) [43]**	**< 0.0001**	**0.40–1.1 (nM)**
PAI-1		24.6 (14.5) [56]	21.3 (14.5) [43]	0.0680	4.0–43.0 (ng/mL)
Factor VIIa		3.5 (1.4) [56]	2.9 (1.8) [43]	0.4312	1.5–4.1 (ng/mL)
PAP complexes		**360.9 (275.8) [56]**	**258.1 (225.8) [43]**	**0.0006**	**80–470 (μg/L)**
P-selectin		**41.8 (40.0) [56]**	**32.1 (21.7) [43]**	**0.0200**	**3–90 (μg/mL)**
ETP		**475.2 (99.2) [56]**	**412.4 (75.8) [43]**	**<0.0001**	**351–473 (mEq)**
Prothrombin time		98.5 (5.0) [56]	98.0 (5.0) [43]	0.4701	0.85–1.15 (ratio)/ 80–100 (%)
Attp		30.0 (3.0) [56]	30.0 (4.0) [43]	0.4849	25–35 (sec)
Fibrinogen		3.5 (0.8) [56]	3.4 (0.8) [43]	0.8540	1.5–4.5 (g/L)
Antithrombin		104.5 (16.5) [56]	101.0 (18.0) [43]	0.4518	60–140 (%)
Plasminogen		108.5 (17.5) [56]	107.0 (16.0) [43]	0.1467	60–140 (%)
Protein C		103.0 (30.5) [56]	104.0 (27.0) [43]	0.7082	60–140 (%)
Total protein S		87.0 (14.5) [56]	88.0 (12.0) [43]	0.8156	60–140 (%)
Free protein S		85.5 (11.5) [56]	88.0 (13.0) [43]	0.1125	60–140 (%)
FVIII		112.5 (51.0) [56]	103.0 (36.0) [43]	0.1391	60–140 (%)
FvWAg		136.0 (52.0) [56]	116.0 (46.0) [43]	0.0758	65–150 (U/dL)
Microparticles		21.1 (11.0) [56]	17.7 (13.5) [43]	0.0112	8–30 (nM)
CD40L		98.2 (39.3) [56]	89.0 (42.1) [43]	0.6952	30–145 (pg/mL)
Tissue factor		116.5 (50.1) [56]	124.2 (57.3) [43]	0.3737	80–280 (ng/mL)
ADAMT13		103.8 (41.9) [56]	97.9 (60.1) [43]	0.8905	50–120 (ng/mL)
Factor XIIa		3.7 (3.9) [56]	3.1 (4.6) [43]	0.3178	1.0–4.4 (ng/mL)
Factor V Leiden ^*b*^	No mutation	55 (98%)	42 (98%)	1.0000 ^*c*^	Mutations/no mutations
	Heterocygote	1 (2%)	1 (2%)		
G20210A^*b*^	No mutation	56 (100%)	43 (100%)		Mutations/no mutations

^a^ Wilcoxon rank sum test *P* value

^b^ Absolute frequency (column percentage)

^c^ Fisher’s exact test

**Fig 1:**
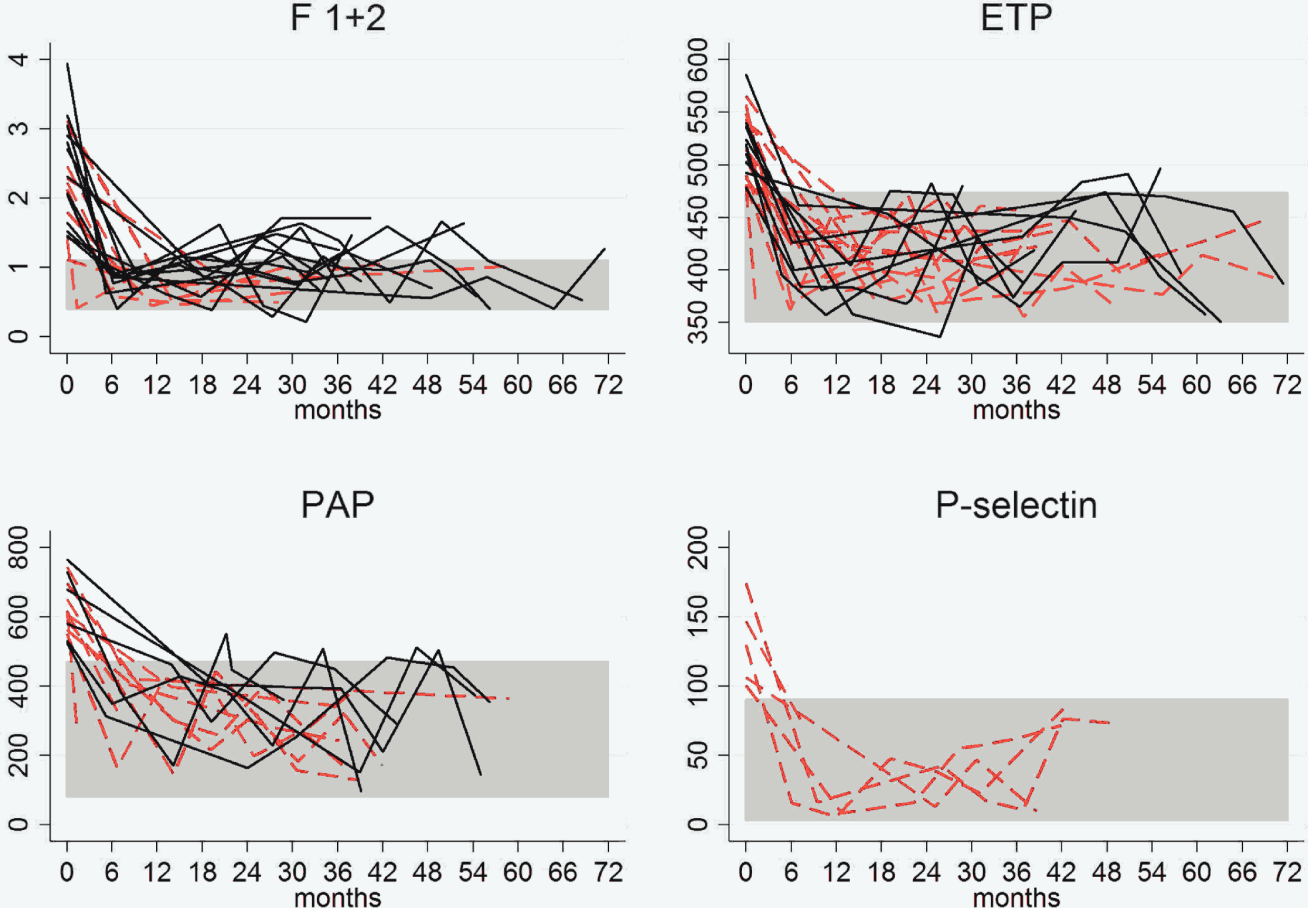
Hemostasis factor levels (baseline and follow-up) in patients with altered levels before treatment who achieved levels within normal ranges during follow-up. *The discontinuous red lines indicate patients who maintained normal values throughout follow-up. The continuous black lines indicate patients who experienced a return to abnormal values at some time during the follow-up. Abbreviations: ETP, endogenous thrombin potential; F 1+2, prothrombin fragment 1+2; PAP, plasmin-antiplasmin.

Thirty-three (76%) of the 43 patients with abnormal baseline F1+2 values achieved normal levels after a median follow-up of 9 month (IQR, 8). All but one of the 28 patients with abnormal ETP values before treatment showed normal values at 6 months (IQR, 3). These values were maintained throughout follow-up (30 months; IQR, 28) in 15 patients (60%). Fifteen of the 17 patients with abnormal baseline PAP values showed normal values 7 months (IQR, 7) after treatment and nine of these (60%) maintained these values throughout follow-up (28 months; IQR,11). However, PAP values at 12 and 48 months seemed to be higher than those at 6 months, but the confidence interval indicates a lack of precision for both time point effect estimates (Table 3). Thus, once normalized, F1+2 and ETP levels did not increase again significantly after treatment. Fig 1 shows a graphic representation of these results.

**Table 3:** Variations in hemostasis F1+2, ETP, and PAP during the follow-up of 50 treated patients.

		F1+2^*b*^		ETP^*b*^		PAP^*b*^	
Variable		Effect Estimate (95% CI)	*P* Value	Effect Estimate (95% CI)	*P* Value	Effect Estimate (95% CI)	*P* Value
TIME	Baseline ^*c*^	0.88 (0.66; 1.10)		52.58 (33.96; 71.21)		101.26 (48.39; 154.14)	
	6 mo	0		0		0	
	12 mo	-0.08 (-0.36; 0.21)		-8.54 (-31.96; 14.88)		65.30 (-1.58; 132.18)	
	18 mo	-0.01 (-0.27; 0.25)	< 0.0001	-6.06 (-27.87; 15.75)	< 0.0001	29.03 (-33.24; 91.29)	< 0.0001
	24 mo	0.03 (-0.25; 0.30)		2.88 (-19.65; 25.41)		-3.97 (-68.14; 60.20)	
	30 mo	-0.10 (-0.39; 0.19)		13.19 (-10.58; 36.95)		49.83 (-17.91; 117.57)	
	36 mo	0.10 (-0.18; 0.37)		0.92 (-21.88; 23.73)		-27.76 (-92.97; 37.45)	

^*b*^ F 1+2, prothrombin fragment 1+2; ETP, endogenous thrombin potential; PAP, plasmin-antiplasmin

^*c*^ Baseline value compared to value at 6 months of follow-up.

F1+2 values are an indirect measure of the amount of thrombin generated in vivo (mainly due to endothelial injury, even in subclinical states) [39], and ETP levels indicate the potential amount of thrombin that can be formed when blood coagulation is activated through the addition of tissue factor. PAP complexes are markers of fibrinolysis. Upon activation, plasmin, which is primarily responsible for a controlled and regulated dissolution of the fibrin polymers into soluble fragments, is immediately inactivated by antiplasmin, forming PAP complexes. [40] Therefore, it is conceivable that the increase formation of PAP complexes stems from excessive formation of fibrin in the blood stream of untreated *T*. *cruzi* infected patients. Soluble P-selectin is considered a biomarker of in vivo platelet activation. P-selectin is contained in the α-granules of platelets; following platelet activation, the soluble form is expressed on the platelet surface and then shed by cleavage. P-selectin has been shown to act as a link between thrombosis and inflammation. [41] Additionally, the four biomarkers-F1+2, ETP, PAP complexes, and P-selectin-reflect are highly stable over time.

A hypercoagulable state is a term that pretends to denominate a condition in which there is an increased tendency toward blood clotting. There is not a universally accepted definition for this state based in biomarkers values, but an increase in several of them suggests the possibility of an increase in the person's chances of developing blood clots. The increases in F1+2, PAP and ETP are congruent with this idea: F1+2 and PAP indicate the actual amount of thrombin and plasmin formed, as markers in procoagulant and fibrinolysis pathways, respectively; and ETP indicates the potential amount of thrombin that can be formed considering globally all the activators, inhibitors and substrates of the hemostasis present in the plasma. The increase observed in these biomarkers is good enough to be an argument to point out a hypercoagulable state in patients with Chagas’ disease.

Sixteen (33%) of the 56 G1 patients had a positive qRT-PCR result at baseline, but only four of these had a positive result after treatment (treatment failure rate of 25% in this subgroup). Five of the 34 patients with a negative baseline qRT-PCR result showed a positive result during follow-up. None of the patients with normal F1+2 values during follow-up had a positive qRT-PCR result, but 3(13%) of the 24 patients with normal ETP values during follow-up did. Of the patients with altered levels of F1+2, ETP, or PAP complexes at baseline, a positive qRT-PCR result during follow-up was not significantly associated with changes observed in lytic anti-α-Gal antibodies, F1+2, ETP, and/or PAP levels.

A positive qRT-PCR result after treatment in patients who achieved normalization of F1+2, ETP, and/or PAP could mean that a decrease in parasite load is sufficient to modify the hypercoagulable state or that benznidazole, which acts on the redox system, could modify these biomarkers without eliminating the parasites. This would limit the use of these factors as biomarkers for parasite elimination, although they could be valuable indicators of treatment response and add support to the theory that, by reverting the hypercoagulable state, benznidazole may also prevent clinical thrombotic events.

Conventional ELISA results were positive in all the patients in G1. Although, as expected, antibodies remained positive throughout follow-up, a slight decrease was detected by the commercial and in-house methods during this period. A statistically significant relevant decrease, was only observed with the in-house test from month 18 onwards (*P* = .0006).

**Fig 2:**
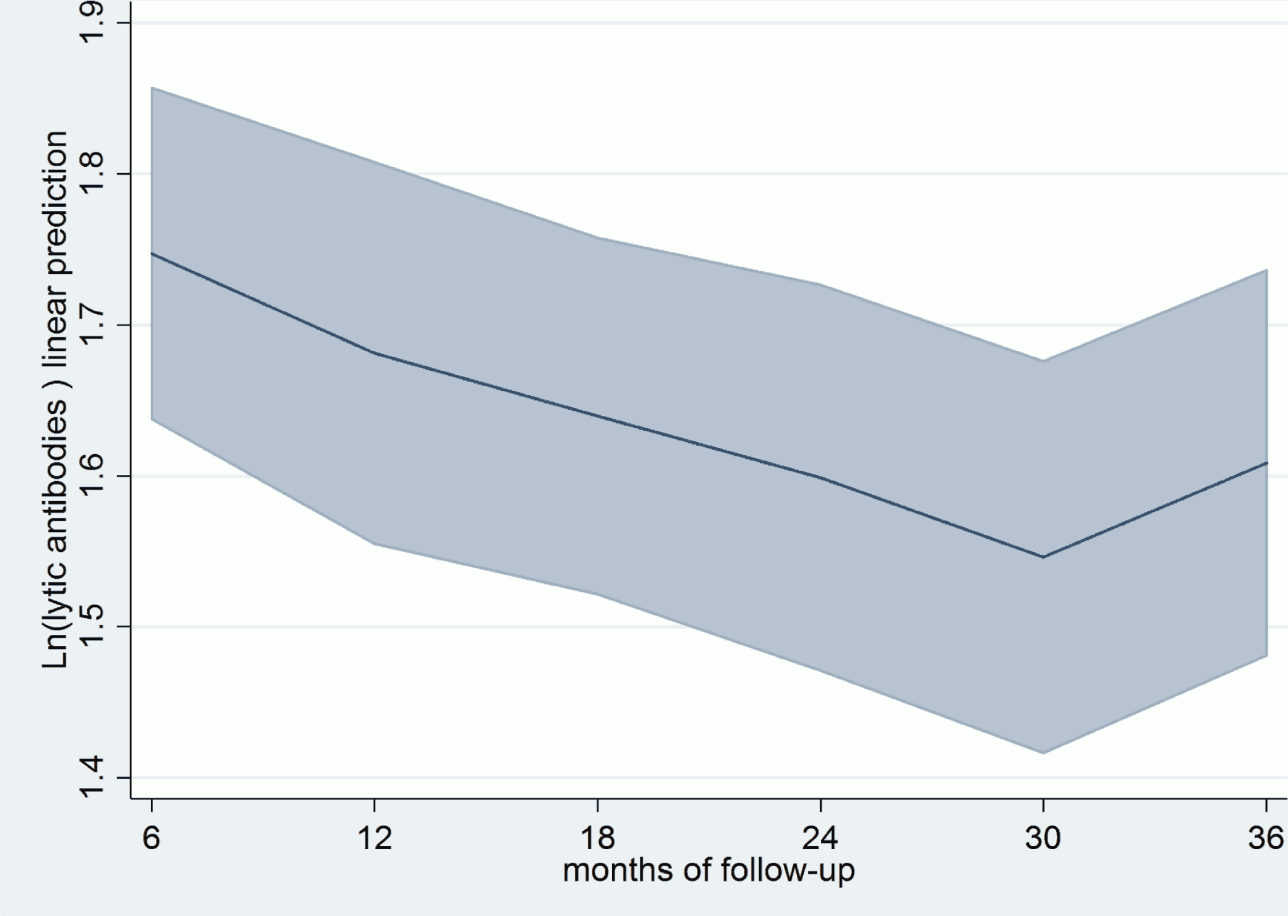
Variations in AT CL-ELISA levels. Months of follow-up predictive margins with 95% CI. Cutoff AT CL ELISA = 0.

Lytic anti-α-Gal antibodies were positive in 52 (96%) of the 54 patients tested before treatment, and in all patients AT CL-ELISA remained within positive levels to the end of the follow-up (Fig 2). Besides, there was no correlation between lytic anti-α-Gal antibody assay and the hemostasis factors evaluated. In relation to previous studies’ results, early decreases in lytic anti-α-Gal antibodies were expected to be observed. On the contrary, a decrease in levels was evident at month 12 and this was significant since month 18 and forward (*P* = .0052). [28, 34]

Adherence to treatment was high, with only five patients not achieving 80% of the total dose. All five patients showed abnormal F1+2 values throughout follow-up and 3 (60%) had abnormal ETP and PAP values. One of the five patients had a positive qRT-PCR result during follow-up, and all five maintained the same positive ELISA and AT CL-ELISA results throughout follow-up. A large cohort of adolescents with *T cruzi* infection treated with benznidazole showed seronegativity in lytic anti-α-Gal antibodies, as measured by AT CL-ELISA, in 58% and 85% of the patients 36 and 72 months after treatment, respectively. [28, 34] The differences between those studies and ours may be due to the nature of the cohorts (adolescents vs. adults) and the stage of the disease. Nevertheless, both studies showed a similar trend towards a reduction in lytic anti-α-Gal antibodies following treatment with benznidazole.

**Fig 3:**
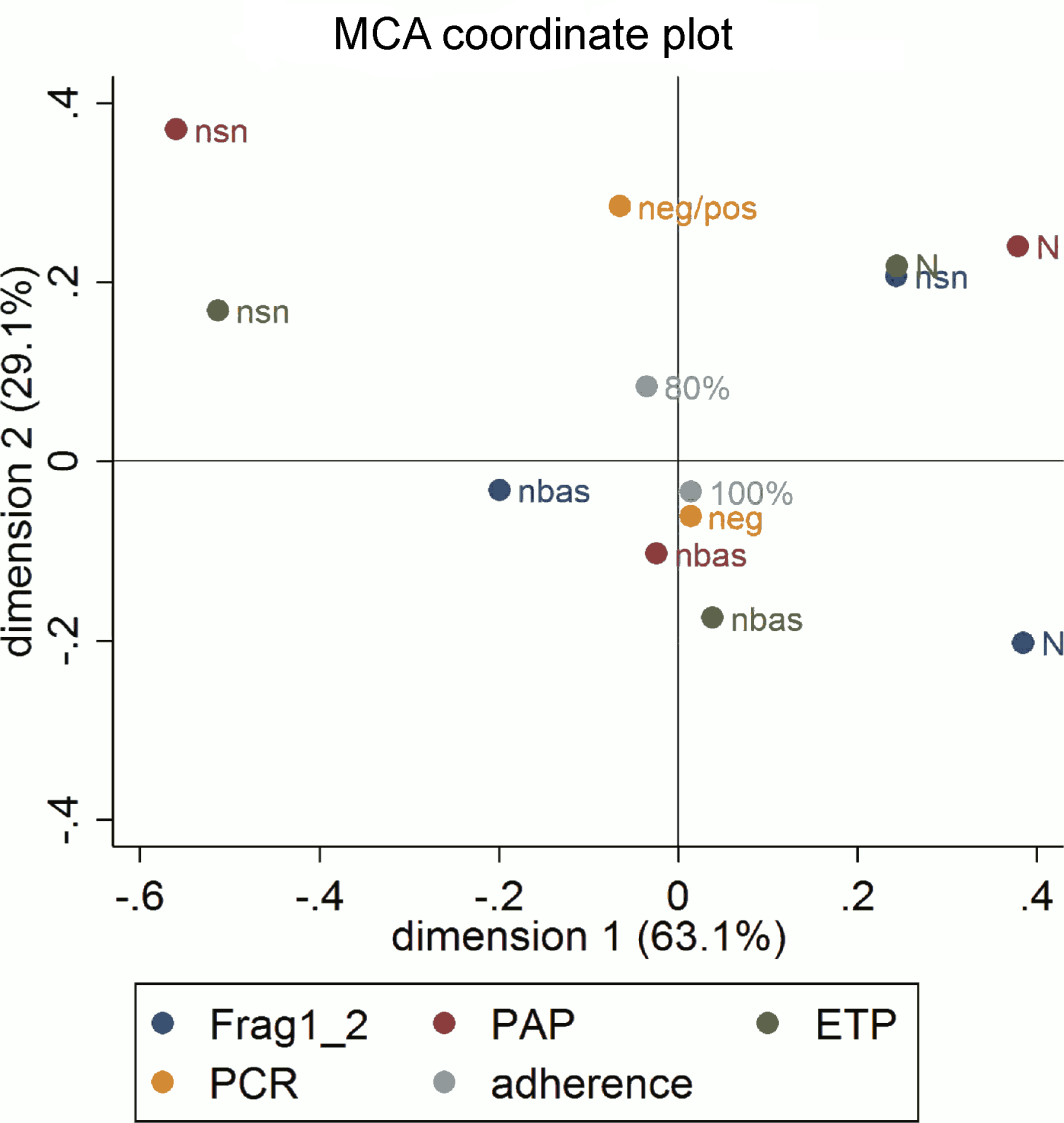
Multiple correspondence analysis coordinate plot. **Biomarkers:** N, sustained normalization of values throughout follow-up; nsn: non-sustained normalization throughout follow-up; nbas, normal value at baseline. Supplementary variables: qRT-PCR during follow-up: neg (negative); pos (positive); Level of adherence: 80%; 100%. Abbreviations: ETP, endogenous thrombin potential; F1+2, prothrombin fragment 1+2; PCR, polymerase chain reaction.

We studied the relationship between normalization of hypercoagulability markers F1+2, PAP, and ETP and qRT-PCR results by multiple correspondence analyses (MCA). Due to the low rate of positive qRT-PCR results, this variable was used as a supplementary variable jointly with treatment adherence. The MCA results (Fig 3) showed an association between complete normalization of PAP and ETP levels and non-sustained and marginally abnormal values in F1+2. These factors had the highest contribution and correlation in the positive part of the second dimension, while normal baseline ETP and PAP values had the highest contribution and correlation in the negative part. F1+2 normalization clearly characterized the positive part of the first dimension, while non-sustained normalization of PAP and ETP values clearly characterized the negative part. In other words, the sustained normalization observed post-treatment in PAP and ETP, could, despite the non-sustained normalization of F1+2 values, reflect response to antiparasitic treatment due to the strong correlation between these three variables.

The projection of qRT-PCR results and adherence to treatment in the solution space provided little additional information. Consistently negative qRT-PCR results throughout follow-up appear to be related to 100% treatment adherence.

In a recent study, the authors found that the serum samples of 37 individuals with chronic Chagas disease showed an upregulation of specific fragments of apolipoprotein A-1 (Apo A1) and one fibronectin fragment, that returned to normal levels in 43% of them three years after a treatment with nifurtimox. [38] Apo A1 and fibronectin fragment were altered in all the 37 patients with *T*.*cruzi* infection before treatment, but the number of patients treated with that normalized levels was lower than in our series (60% and 96% of patients who normalized F1+2 and ETP values).

This study has some limitations. Although the sample size was calculated to obtain sufficient statistical power to answer the hypothesis, a larger sample may have detected differences that would be expected to appear earlier (e.g., before 12 months). The lost to follow-up samples also affected the estimates. Even within Spain, it is difficult to follow individuals with high migratory mobility for long periods. In addition, the fact that only 30% of patients had a positive baseline qRT-PCR result was a constraint for assessing the effect of treatment.

In conclusion, patients with chronic *T*.*cruzi* infection have a potential hypercoagulable state, regardless of cardiological and/or digestive involvement. The hypercoagulability markers F1+2 and ETP were abnormally expressed in a high percentage of patients with chronic *T*.*cruzi* infection before treatment (77% and 50%, respectively) but returned to and remained at normal levels shortly after treatment in 76% and 96% of patients, respectively. Baseline PAP values were altered in just 30% of patients before treatment, but normalized several months after treatment in 88% of these. These three hypercoagulability biomarkers could be useful for assessing short-term response to treatment. However, the fact that normal values were seen in some infected patients, including some with positive post-treatment qRT-PCR results, reduces their usefulness as universal biomarkers. The decrease in hypercoagulability factor levels could be explained by a decrease in parasitemia or by other benznidazole effect.
